# Exposure to marketing of breastmilk substitutes in Mexican women: Sources and scope

**DOI:** 10.1186/s13006-022-00455-y

**Published:** 2022-03-02

**Authors:** Sonia Hernández-Cordero, Mireya Vilar-Compte, Ana Cristina Castañeda-Márquez, Nigel Rollins, Gillian Kingston, Rafael Pérez-Escamilla

**Affiliations:** 1 Research Center for Equitable Development EQUIDE, Universidad Iberoamericana, Prolongación Paseo de la Reforma 880, Lomas de Santa Fe, 01219 Mexico City, Mexico; 2 Department of Public Health, Montclair State University, 1 Normal Avenue, Montclair, NJ 07043 USA; 3 School of Public Health of Mexico, National Institute of Public Health, Av. Universidad 655, Cuernavaca, Morelos, México; 4 Department of Maternal, Newborn, Child and Adolescent Health, World Health Organization, Avenue Appia 20, 1202 Geneve, Switzerland; 5 Executive Fellow, School of Business, Kings College London, London, England; 6 Department of Social and Behavioral Sciences, Yale University School of Public Health, New Haven, 06510 CT USA

**Keywords:** Breastmilk substitutes, Promotion, Health providers, Infant feeding practices, Mexico, International Code of Marketing of Breast-milk Substitutes, Regulation

## Abstract

**Background:**

Aggressive and unregulated marketing of breastmilk substitutes (BMS) results in increased child morbidity and mortality. Unregulated BMS marketing is a major public health concern because it encourages formula consumption at the expense of breastfeeding. This study aimed to identify the sources and characterize the nature of exposure to marketing of BMS among Mexican mothers of children under 18 months of age. As a secondary objective we explored potential association between exposure to BMS marketing and infant feeding practices.

**Methods:**

Cross-sectional study, comprising a pre-piloted survey, was conducted between February 2020 to February 2021 with Mexican mothers of children under 18 months of age (*n* = 754), in two major cities in Mexico. Mothers were selected according to their current infant feeding practices (Breastfeeding only vs. Mixed feeding). We characterized the different BMS marketing sources and scope, and related them with infant feeding practices. In addition, we used logistic regression models to estimate the odds ratio for infant feeding practices by BMS marketing exposure or recommendation.

**Results:**

Mothers reported different sources of exposure to BMS promotion, including BMS advertisements in diverse media channels (41.6%), recommendation by a healthcare professional and/or relative (76.2%), and receiving a BMS sample at a hospital (18.6%). By contrast, only 36.5% recalled hearing or seeing breastfeeding information the previous year. The odds of mixed feeding were substantially higher, compared to breastfeeding, when mothers were recommended to use a BMS by doctors/pediatricians (OR: 3.96, 95% CI: 2.00, 7.83). Having seen or heard breastfeeding information in the previous year was associated with a lower risk of mixed feeding compared to breastfeeding only (OR: 0.59, 95% CI: 0.35, 0.99).

**Conclusions:**

Mexican mothers of young children in the metropolitan areas studied were highly exposed to BMS marketing and through different mass media channels and inter-personal sources. Health care professionals, particularly doctors/pediatricians, are a source of BMS promotion that are likely to have a strong influence on maternal decisions about infant feeding practices. There is an urgent need to protect mothers and their families against unregulated BMS promotion through mass media channels and directly by influential individuals, including health care providers.

**Supplementary Information:**

The online version contains supplementary material available at 10.1186/s13006-022-00455-y.

## Background

The contribution of breastfeeding to child survival, health, and development as well as to the improved mother's health, and national development has been well documented [1]. Globally, breastfeeding practices are suboptimal around the world. The World Health Organization (WHO) recommends early breastfeeding initiation (within the first 60 min post-partum), exclusive breastfeeding for 6 months and continuation of breastfeeding until at least 2 years of age once complementary foods are introduced [2]. In Mexico, in 2018 less than 50% of newborns were put to the breast within 60 min postpartum, the rate of exclusive breastfeeding in infants aged < 6 months was 28.6%, and only 46.9% and 36.9% continued breastfeeding at 1 and 2 years, respectively [3].

Mothers' decisions on infant feeding practices are influenced by socio‐economic, cultural, and individual factors, including the marketing from the breastmilk substitutes (BMS) industry [4, 5]. BMS include any milks or complementary foods (or products that could be used to replace milk, such as fortified soy milk), in either liquid or powdered form that are specifically marketed for feeding infants and young children up to the age of 3 years. It includes infant formula (for infants 0–6 months), follow‐up formula (for infants 6–12 months), growing‐up milk (children 12–36 months) and complementary foods (6–36 months) [6]. Promotion of BMS negatively affects the choice and ability of mothers to optimally breastfeed their children [4].

Infant formula sales continue to increase. The retail value of the infant formula market which was estimated at US$44.8 billion in 2014, was predicted to increase to US$70.6 billion by 2019 [4]. Globally, infant formula sales grew by 40% in the period 2008–2013 [7], mainly due to infant (defined as breastmilk substitutes designed to fulfill the requirements of an infant during the first months of life) [8] and follow-up formulas (a liquid prepared from the milk of cows or other animals and/or other constituents of animal and/or plant origin proteins intended to be part of the weaning diet of young children 6–12 months) [8].

It has been well documented that the aggressive and unregulated marketing of BMS results in increased child morbidity and mortality, especially in low- and middle-income countries [9], the importance of breastfeeding protection and promotion in high-income countries is clearly understood [1]. Marketing, as defined by WHO in its “Guidance on ending the inappropriate promotion of foods for infants and young children”, includes “product promotion, distribution, selling, advertising, as well as direct contact of the BMS producers with mothers, and information services” [10]. BMS marketing has used different channels and strategies, such as TV and radio mass media, printed media, company web sites, social media (e.g., twitter, Facebook), incentives such as free product samples that can be obtained from retailers at point of sales and online, promotions to and through health facilities, health care professionals and policy makers. Unregulated BMS marketing is a major public health concern because it encourages formula consumption at the expense of breastfeeding [11].

Globally, it has been documented that mothers are heavily exposed to BMS promotion [12–15], and that this exposure influences their infant feeding decision [12, 16–18]. In Mexico, this is also true, where it has been reported that Mexican women from two large cities received BMS promotion through health services, point of sales, BMS products’ labels (mainly as nutrition and health claims), TV and internet [13, 14]. However, more research is needed on the range of BMS promotion channels and strategies to reach Mexican mothers, including social media and other potential sources and its potential association with infant feeding decisions.

## Methods

### Aim

To identify the sources and characterize the nature of exposure to marketing of BMS among Mexican mothers of children under 18 months of age. As a secondary objective we explored potential association between exposure to BMS marketing and infant feeding practices.

### Design

Cross-sectional study. This study is part of a multi-country study, Mexico being one of them. The study drew on 8 data collection methods to map the marketing of formula milk, including qualitative and quantitative methodologies. The overall aim of the primary study was to document the reach of formula milk marketing, how formula milk marketing messages are perceived by women and influencers, and their effect on knowledge and values. The present paper describes the results of a pre-piloted quantitative survey, conducted face-to-face with Mexican mothers of children under 18 months of age.

### Settings

The study took place in Mexico City and Guadalajara, the second and third, respectively, largest metropolitan areas in the country. In both cities, 31% of their population is made up of women in reproductive age, and a percentage of more than 70% of the population with access to health services [19, 20]. The study was conducted between February 2020 to February 2021. The protocol was approved by the ethics committee of the World Health Organization (WHO) and by the Research Ethics Committee of the National Institute of Public Health (Comité de Ética en Investigación, Instituto Nacional de Salud Pública). Informed consent was obtained from all study participants.

### Sample

Mothers of children under 18 months of age were selected for this study based on the size and heterogeneity of populations across neighbourhoods and households. The sampling framework was designed to achieve representativeness of low, medium–low, medium, and high socioeconomic status (SES) groups; child’s age, and infant feeding practices. M&C Saatchi World Services, together with their local Mexico partners, INSAD (Investigación en Salud y Demografía, S.C) selected public and private health care units selected to recruit the sample were those which provided the most services to pregnant women and mothers localities through convenience sampling to ensure the inclusion of the before mentioned SES groups. SES was assigned using the socioeconomic status from the “Agencias de Inteligencia de Mercado y Opinión Pública” (AMAI), 2018 [21]. The AMAI´s SES classifies households into seven levels, considering six characteristics of the household (schooling of the head of household, number of bedrooms, number of complete bathrooms, number of employed persons aged 14 and over, number of cars, vans and vans, and fixed internet access in the home). The six levels are classified as (going from very low to very high): E, D, D + , C-, C, C + , A/B. For our study, the convenience sampling was designed to include mothers from the SES of low and medium–low (D and D +), medium and medium high (C- and C), as well as high and very high (C + and A/B). The public health care units (part of the Mexican Ministry of Health) mainly provided care to people within SES groups, which represent low and medium–low SES while people who were in the medium and high SES groups, typically went to the private health care units. Hospitals were also included to represent low and medium SES groups. Selection of participants within clinics/health facilities was via convenience methods.

### Recruitment and inclusion/exclusion criteria

Participants were recruited from public and private hospitals and clinics in Mexico City and Guadalajara. However, once the COVID-19 pandemic started, access to hospitals was limited and other recruitment sources were needed including squares and public areas near health facilities. To participate in the study, mothers needed to be over 18 years of age and have a child under 18 months of age at the time of the interview. Potential participants were excluded if the mother or the baby had a pathology that could limit breastfeeding, or herself or a relative worked at a BMS company or related industries. In total 754 mothers with children aged 0–18 months were included in this study. A convenience sampling method was employed to recruit participants following a quota sampling approach by infant feeding practices (breastfeeding or BMS feeding), child's age (0–12 months for breastfed infants, 0–3 months, 4–6 months, 7–12 months, 13–18 months for BMS fed) and SES group (low, medium and high) of 25 women on each category (Additional file 1- Survey sample). The SES selection was based on the Water/Sanitation, Assets, Maternal Education and Income (WAMI) index. This index is a multicounty validated SES index which assesses access to improved water and sanitation, and selected assets and home characteristics (number of bathrooms in their home, number of cars/vans they have, access to the internet, number of working people in home aged 14 years old or more, number of bedrooms), maternal education, and household income [22].

### Data collection

The pre-piloted survey was conducted face-to-face between February and May 2020, by standardized personnel, using a tablet with Computer Assisted Personal Interviewing software (CAPI).

### Definition of variables

#### Exposure to BMS marketing

Assessed, based on mothers’ response to the question: *“In which of these locations have you frequently seen [in the previous year] any type of marketing or advertising for formula brands?”* The three categories used in this analysis were: Social media (including YouTube, Facebook, Instagram and Mothers Club online), TV or radio, and Other (including Company´s website, e-mail, Health Care, Hospitals or Clinics, magazines, newspaper, billboard, supermarket or adds in an elevator). Respondents were asked in the survey what type of formula (i.e. infant or follow-on formula, growing-up milk) they saw advertising for.

#### BMS use recommendation

To access this variable, women were asked *“Have any of these individuals or groups ever recommended that you should feed your baby formula milk?”* and a list of answer options were provided. For the analysis, the following categories were considered: Nutritionist/nurse; Doctor/Pediatrician; Relative/Friend, Other, None of these.

#### BMS exposure promotion index

The index was calculated by adding the BMS marketing contact and or exposure, which included exposure to BMS advertisement through different sources in the previous year. The sources considered for the index included BMS adverts through traditional media (TV, radio, magazines, newspaper, billboards), digital media (YouTube, companies or professional websites, social media) email, health facilities, supermarkets (online and physical), free samples in hospitals and direct contact with BMS companies. The index considered the different types of sources only, it did not consider frequency as it was not advised to attempt to measure recall of frequency of exposure. The index had a range from 0–14. We included it in the analysis, as a continuous and as a 4-level categorical variable (No exposure; Score 1–2, Score 3–5; Score 6 or more) or binary variable (No exposure; and Exposure with at least one reported marketing touchpoint).

#### Infant feeding practices

A binary categorical variable was used to model current infant feeding practices. Mothers were asked to answer the question: *“How are you currently feeding your youngest baby?”*. Based on the responses, we ended up with 2 infant feeding categories: “Breastfeeding only”, i.e. children consuming breast milk only; “Mixed feeding”, children who consumed both breastmilk and BMS as well as BMS only.

#### Perceived benefits of BMS

This information was obtained with the question: *“What do you think are the benefits, if any, of using formula milk?”* Mothers were allowed to ascertain more than one perceived benefit either for the mother or the baby. The ascertainment options included: allows me to leave the baby with someone else; allows me to do other activities/to get back to work; the baby is calmer; it’s a quick option; the baby improves his/her health; the couple is involved in feeding the baby; it is an option when you do not have milk; among others.

We grouped the answers provided by the mothers into the following categories Additional file 2- Perceived BMS benefits categories): Convenient for mother and family; Perceived benefits for the baby; Needed when insufficient milk; None; Do not known.

#### Seeing or hearing breastfeeding (BF) information in the previous year

We considered that women received BF information, if women reported having seen or heard any advertising about breastfeeding in the past year *(“In the past year have you seen or heard any advertising about breastfeeding?”* This was kept as a dichotomous variable (i.e. yes/no)).

#### Previous experience with BF

Represented by a binary categorical variable, indicating experiences with breastfeeding or BMS feeding with a prior child.

##### ***Other variables***

All statistical models were adjusted for maternal employment status ( “Yes” included those reporting: full time job, partial time job, maternity leave, Student; “No” included mothers reporting household chores, unemployed or looking for a job).; maternal age (as continuous variable); maternal education level (Secondary or less, highschool and undergraduate and graduate); type of hospital where delivery took place (Public hospital; Private hospital; Don’t know or not sure); and infants’ age (as reported by the mother): 0- 5 months, 6–11 months, and 12–18 months).

### Data analysis (statistics)

We estimated descriptive statistics, and conducted chi-square and U-Mann–Whitney analyses to describe the different BMS marketing sources and scope, and with infant feeding practices. As an additional analysis, we used logistic regression models to estimate the odds ratio for infant feeding practices by BMS marketing exposure or recommendation. The significance level was considered with *p* < 0.05. All analysis was performed stratifying by child's age (infants < 5 months, 6–11 months, and children 12–18 months). No differences were found on the stratified analysis, thus unstratified analysis were presented. All analyses were conducted in Stata v14.1 (StataCorp, College Station, TX, USA).

## Results

The average age of the mothers participating in the study was 27.6 (± 6.3) years. Most of the mothers (69.4%) were not employed at the time of the interview, and almost 52% had an education level of secondary or less. Most mothers (55.0%) had at least one child at the time of the study. For the great majority of indicators there were no sociodemographic differences by infant feeding practices. Two exceptions were age and employment status; those who were breastfeeding only were slightly younger (26.1 years) and less likely to be working (81.5% of breastfeeding only mothers were not working at the time of the study) compared to women in the mixed feeding group (age 27.9 years old and 66.3% were not working) (*p* < 0.05) (Table 1).

**Table 1:** Characteristics Mexican mothers with children < 18 months participating in the study (*n* = 754)

	**Infant feeding practices**			
	Breastfeeding only (*n* = 151)	Mixed feeding (*n* = 603)	Total (*n* = 754)	*p-value* ^a^
**Age (years)**^**b**^	26.13 ± 5.7	27.9 ± 6.5	27.6 ± 6.3	**0.002**
**Parity- n (%) **^**c**^				
Primiparous	68 (45)	257 (42.6)	325 (43.1)	0.59
Multiparous	83 (55)	346 (57.4)	429 (56.9)	
**Education level—n (%) **^**c**^				
Secondary or less	78 (51.7)	302 (50.1)	380 (50.4)	0.22
High school	61 (40.4)	223 (37)	284 (37.7)	
Undergraduate and graduate	12 (7.9)	78 (12.9)	90 (11.9)	
**Employment status- n (%)**^**c**^				
Yes	28 (18.5)	203 (33.7)	231 (30.6)	** < 0.0001**
No	123 (81.5)	400 (66.3)	523 (69.4)	
**Child´s age- n (%) c**				
One month or younger	21 (13.9)	33 (5.4)	54 (7.2)	** < 0.0001**
2—3 months	27 (17.9)	117 (19.4)	144 (19.1)	
4—6 months	36 (23.8)	150 (24.9)	186 (24.7)	
7—9 months	32 (21.2)	65 (10.8)	97 (12.8)	
10—12 months	35 (23.2)	85 (14.1)	120 (15.9)	
13—18 months	0 (0)	153 (25.4)	153 (20.3)	
**Previous experience breastfeeding – n (%) **^**c**^				
Yes	76 (91.6)	295 (85.3)	371 (86.5)	0.13
No	7 (8.4)	51 (14.7)	58 (13.5)	

^a^*p* < 0.05, ^b^ANOVA test, ^c^Chi-Squared tests

**Fig. 1:**
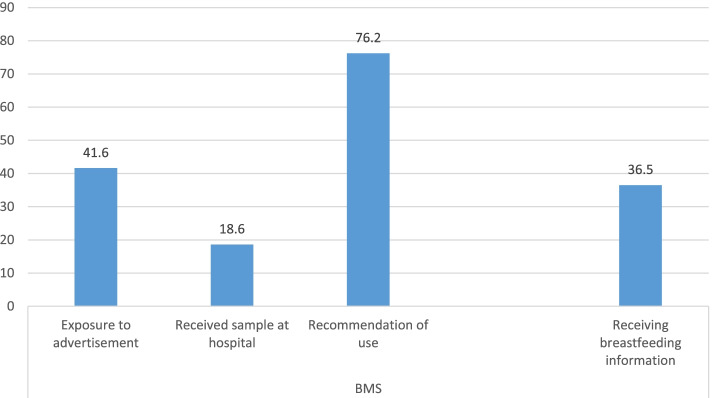
Exposure to BMS (BMS: Breastmilk substitutes) promotion and to breastfeeding information (percentage) among Mexican women participating in the study (*n* = 754)

**Fig. 2:**
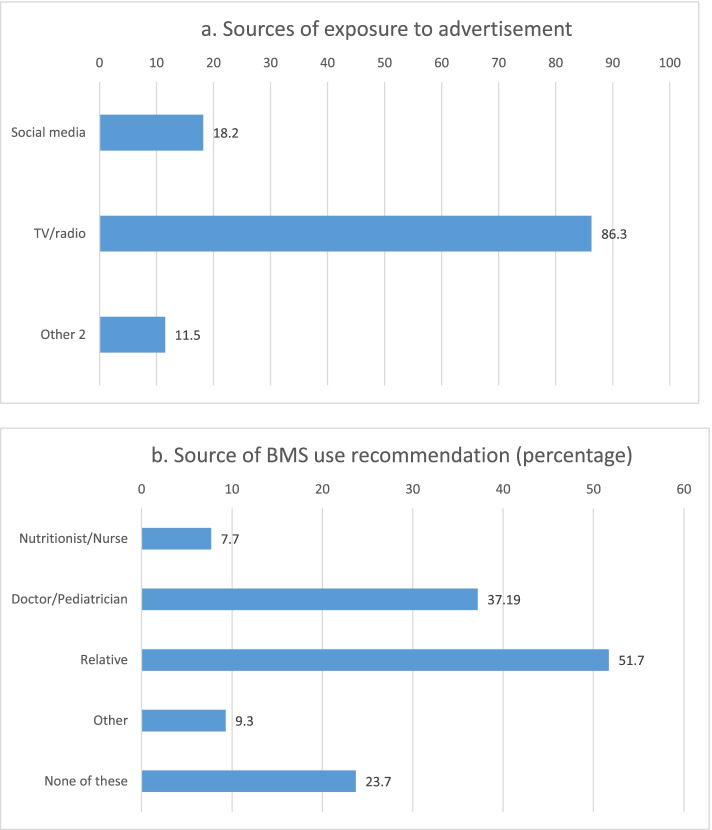
Source of exposure to advertisement and BMS (BMS: Breastmilk substitutes; ^1^ Supermarket, billboard, health center, magazine, company website use recommendation (percentage) among Mexican women participating in the study (*n* = 754). **a** Sources of exposure to advertisement. **b** Source of BMS use recommendation

Mothers reported multiple sources of exposure to BMS promotion, including BMS advertisement in any media channel (41.6%), BMS use recommendation by a health care professional and relatives (76.2%) and receiving a BMS sample at a hospital (18.6%) (Fig. 1). Traditional media (TV and radio) was the most common source of BMS promotion exposure (86.3%), followed by social media (18.2%) (Fig. 2.a). They reported relatives and health care professionals as the main source of BMS use recommendation (Fig. 2.b). As for exposure to BF information, only 36.5% recalled seeing or hearing BF information the previous year (Fig. 1), with TV being the most common source (53.8%), followed by primary health facilities (17.1%), hospitals (16.0%), social media (13.8%) and radio (3.6%).

According to the marketing score, 60% of participants reported being exposed to at least one source of BMS promotion and marketing, with 22.2% of them, reporting 3 or more sources of exposure (Table 2).

**Table 2:** Marketing score and infant feeding practices among Mexican mothers with children < 18 months participating in the study (*n* = 754)

	Infant feeding practice			
	Breastfeeding only, *n* = 151	Mixed feeding^a^, *n* = 603	Total, *n* = 754	*p-value*^b^
**Marketing score**				
No exposure	60 (39.7)	238 (39.5)	298 (39.5)	
Score 1–2	53 (35.1)	236 (39.1)	289 (38.3)	
Score 3–5	30 (19.9)	101 (16.8)	131 (17.4)	0.69
Score 6 or more	8 (5.3)	28 (4.6)	36 (4.8)	
**Marketing score**				
No exposure	60 (39.7)	238 (39.5)	298 (39.5)	
Exposure	91 (60.3)	365 (60.5)	456 (60.5)	0.99

^a^ Mixed feeding: Includes children receiving infant formula only and breastfeeding and formula feeding; ^b^Chi-Squared tests

There was no association between the marketing score and infant feeding practices, even after controlling for potential confounders (data not shown). However, being exposed to BMS promotion was associated with reporting a perceived BMS benefit. Specifically, mothers exposed to BMS promotion reported more frequently as a BMS benefit “Needed when insufficient milk” (49.6%) compared to those not being exposed (42.3%) (*p* = 0.03). This relationship was statistically insignificant after adjusting for maternal and child´s characteristics (OR 1.38, 95% CI: 0.93–2.07) (Data not shown).

There were greater odds of mixed feeding compared to breastfeeding only among women receiving a BMS use recommendation from health care professionals (OR: 3.96, 95% CI: 2.00–7.83). By contrast, seeing or hearing BF information in the previous year was associated with a lower risk of mixed feeding compared to breastfeeding only (*p* = 0.04) (Table 3).

**Table 3:** BMS use recommendation, breastfeeding promotion and infant feeding practices among Mexican mothers with children < 18 months participating in the study (*n* = 428)^1^

	**Mixed feeding**^**b**^		
	**OR**	**95% CI**	*p-value*
**Recommendation of BMS**			
** Nutritionist/Nurse**^**e**^			
Yes	0.96	0.34—2.70	0.94
** Doctor//Paediatrician**^**e**^			
Yes	3.96	2.00—7.83	** < 0.0001**
** Relative/Friend**^**c,d**^			
Yes	1.69	1.00—2.87	**0.04**
**Seeing or hearing Breastfeeding Information**^**d**^			
Yes	0.59	0.35–0.99	**0.04**

^a^ Logistic regression model; ^b^ Reference: Breastfeeding only; ^c^ Mother, mother in law, partner, friend. ^d^Adjusted by Employment status, Mother's, age, Education level, Previous experience feeding baby, child's age; ^e^Adjusted by Employment status, Mother's age, Education level, Previous experience feeding baby, birth hospital, child's age

## Discussion

This study identified a high level of exposure to BMS promotion among Mexican mothers of children under 18 months, through multiple sources. The different sources reported by the mother were direct BMS recommendation by health care professionals and relatives, advertisement through different media (TV, radio or social media) as well as receiving BMS samples at a hospital. The findings of this study support other evidence that BMS promotion undermines breastfeeding according to current recommendations [6, 11, 12] and how suboptimal breastfeeding results in almost 600,000 childhood (6–59 months) deaths each year and adequate breastfeeding have the potential to prevent 98,243 maternal deaths from breast and ovarian cancers as well as type II diabetes annually [23] highlights the implications of these findings. The exposure to BMS promotion among Mexican mothers with children younger than 24 months had been documented before. In a study aimed at estimating the prevalence of violations of the International Code of Marketing of Breast‐milk Substitutes (Code) in two cities of Mexico, women attending public and private health facilities reported receiving free samples of infant formula (11%) and other BMS products in health facilities; and more than 80% of participants recalled seeing BMS publicity in mass media and 3% within health facilities [14]. In another study, that conducted a 2 week- monitoring of the programming of four main Mexican TV channels at peak times to identify BMS advertisements, it was concluded that the Mexican population is frequently exposed to BMS advertisements that breaches the Code, on the internet, on social media, and on television [13]. Although Mexican legislation bans the promotion of BMS (i.e. formula for children younger than 12 months), in our study women reported having been exposed through TV, radio and social media advertising, as well through health services, either by directly receiving a BMS sample or by a health provider recommendation.

Our results show that health care professionals are an important source of BMS promotion that are likely to have a strong influence on maternal decisions about infant feeding practices; the study finds that women who received a recommendation to use BMS by a health care professional were four times more likely to practice mixed feeding than only BF. These findings are highly consistent with previous research in Cambodia and Nepal, where there was also a strong association between health worker recommendations of BMS and BMS feeding [12]. Health care professionals around the world are seen as a credible source of information, thus when the BMS recommendation comes from them, this becomes a strong endorsement of these commercial products [11]. Even though the Mexican legislation states that routine use and promotion of infant formula should be avoided within health facilities [24], we identified that receiving a BMS sample at a hospital is a common source of BMS promotion exposure. This marketing strategy, which violates the Code, and the Mexican legislation as well, has been shown to undermine duration of exclusive breastfeeding, probably because it presumes an endorsement of infant formula by health facilities and health staff [25–27]. The health professional’s recommendation to mothers to feed BMS is a major public health concern, regardless if it is a result of lack of knowledge of breastfeeding benefits, or as a result of a close interaction of providers with BMS industry representatives. Moving forward it is of paramount importance to strengthen the ability of health care systems to protect, promote and support breastfeeding by improving the training of health providers on breastfeeding and, specifically, by addressing conflict of interest. More research is needed to understand the main drivers for health personnel recommending BMS in Mexico. In addition, designing and enforcing a strong regulatory framework to avoid BMS promotion by health care professionals and other Code violations is needed.

Another important finding in this study is the association between recommendations of a relative or friend to use BMS and the increase in the likelihood of mixed feeding. The recommendations of a relative or friend to use BMS is a practice that has been documented previously [18], but not its relation to infant feeding practices decisions. This might represent a level of cultural embeddedness of BMS that adds to that coming from recommendation of BMS by health care professionals and women receiving BMS samples at health facilities [14].

We did not find an association between the marketing score and infant feeding practices, as reported by others [17]. However, this score was associated with perceived BMS benefits, which might be explained by the influence of marketing on enforcing social norms, in this instance making BMS a socially acceptable. Strategies to market BMS have focused on presenting their products as the solution to mothers' concerns about breastfeeding failure, spitting up, fussiness and other “expected” infant behaviors, as described by Piwoz and Huffman [11]. One potential reason for the lack of association between BMS marketing and infant feeding practices in our study is that BMS marketing, which is widespread in Mexico [13, 14], was less influential in decisions around feeding BMS than recommendations from health professionals. Furthermore, the results of this study illustrate the complexity of maternal infant feeding decisions and how breastfeeding and regulating BMS marketing, as it has been described by Barker and colleagues [28] and hence the difficulties of studying associations between exposures to marketing and infant feeding practices. Finally, an alternative explanation for the lack of association between infant feeding practices and marketing exposure is well documented structural barriers to breastfeeding in Mexico, such as barriers within the health system (maternal and child hospital care practices, including use of infant formula to feed newborns, BMS promotion within the hospital, lack of support to women to initiate breastfeeding, and births by cesarean section), in the community (beliefs and perceptions about milk insufficiency and common difficulties faced during breastfeeding, as well the recommendation of BMS used by family members) [18] as well as lack of adequate legal protection for breastfeeding among women working in the formal sector [29].

Our findings also showed that recalled exposure to breastfeeding information (either recalling seeing or hearing about breastfeeding) in the previous year was associated with greater odds of breastfeeding, even among women who had also been heavily exposed to breastmilk substitutes marketing. Even though, in our study we did not identify the specific type of breastfeeding information received, prior studies have shown that breastfeeding counselling or education can improve breastfeeding practices [30]. It is well known that unregulated marketing through mass media channels and direct promotion of BMS undermine the efforts and investments of breastfeeding and young child feeding programs.

BMS producers invest large amounts of money on marketing. It has been estimated that the US Mead Johnson Nutrition company, spent $223.8 million in 2016 marketing BMS and children's nutrition products to both parents and health care in Latin America, Asia, Europe and North America, including advertising on TV, print, and other consumer media, including social media [31]. By contrast, investment on different policies and interventions to promote, protect and support breastfeeding has been quite modest and is declining [32].

According to the 2020 Status of the Code report [33], Mexico´s legislation is “moderately aligned with the Code”, with low scores for monitoring and enforcement (score of 3 out of 10) information and education materials (0/10), and engagement with health workers and systems (5/10). In addtion, there is still an urgent need to update Mexican regulations regarding complementary foodss for infants and young children; as stated in the 2016 WHA 69.9 resolution [6]. The effort to update Mexican regulations to include ultra-processed foods for infants and young children, should be part of other initiatives contributing to obesity prevention in Mexico, such as taxing unhealthy food products, regulating the school environment, and adopting front-of-pack warning labels [28, 34].

In this study the exposure to marketing was measured across multiple survey indicators. The primary indicator was a self-reported exposure to marketing in the past year. The participants were also asked separate questions about the types of marketing that they were exposed to, such as free samples of formula, or invitations to a baby club. The primary indicator was based on a previous study by Sobel and colleagues [35], which measured exposure to marketing through self-reported exposure to marketing. Whilst there is a growing literature on the extent and influence of the marketing of breastmilk substitutes, there are still a limited number of studies which quantitatively measure exposure to, and the influence of marketing at the personal level, which represent a strength of this paper. A one-year timeframe for measuring exposure to marketing was chosen because, to our knowledge, there is no standardized time frame for measuring it. Other methodologies, exploring maternal exposure to BMS marketing, such as the Network for Global Monitoring and Support for Implementation of the International Code of Marketing of Breast-milk Substitutes and Subsequent relevant World Health Assembly Resolutions (NetCode), uses a six-months time frame. However, it is hard to quantify the most accurate time frame for recall as it is dependent on the individual experience. The participants of this study had a child aged 0–18 months thus a one-year time frame was chosen to account for periods of confinement that women may experience such as in hospital, or due to cultural norms, thus it might represent a wider view of the time of exposure to marketing.

The study had some limitations, first it is observational preventing causal inferences. Second, purposive sampling was used and the study was only conducted in two large cities of the country; hence, the generalizability of findings should be interpreted with caution. However, even though we have a non-probabilistic study design, selection bias in the study might be limited by using sampling quotas on infant feeding practices, child's age and SES. In addition, we adjusted the statistical analysis for several factors such as mothers' socioeconomic status, feeding experience, use of public or private sector health services and child´s age. Some residual selection bias may have occurred because of convenience sampling and a lack of breastfed only infants over 12 months old.

## Conclusion

Mexican mothers of children under 18 months were highly exposed to BMS marketing and through different mass media channels and individual sources. This indicates that the Code is not being properly enforced in spite of having been already adopted through legislation. There is an urgent need to protect mothers and their families against unregulated BMS promotion through mass media channels and in the health system, including directly by health care professionals. Mothers have the right to accurate, unbiased information to make an informed decision about their infant feeding choices. Because the vast majority of mothers in Mexico are choosing to breastfeed [3, 36], it is essential to invest in strengthening the environments needed [4, 5] for them to implement their right to breastfeed for as long as recommended.

## Supplementary Information

**Additional file 1:** Quota’s survey sample by infant feeding practices, infant´s age, socioeconomic status and city.

**Additional file 2:** Perceived BMS benefits categories.

## Data Availability

The data that support the findings of this study are available from WHO but restrictions apply to the availability of these data, which were used under license for the current study, and so are not publicly available. Data are however available from the authors upon reasonable request and with permission.

## Abbreviations

AMAI: Asociación Mexicana de Agencias de Inteligencia de Mercado y Opinión
BF: Breastfeeding
BMS: Breastmilk substitutes
CAPI: Computer Assisted Personal Interviewing
INSAD: Investigación en Salud y Demografía
SES: Socioeconomic status
WAMI index: Water/Sanitation, Assets, Maternal Education and Income index software
WHO: World Health Organization
