# Supplementary material for: Exposure to marketing of breastmilk substitutes in Mexican women: Sources and scope
Source: Int Breastfeed J. 2022 Mar 2;17:16. doi: 10.1186/s13006-022-00455-y (PMC8889386; doi:10.1186/s13006-022-00455-y)
Supplement: Supplementary file 1 — Additional file 1: Quota’s survey sample by infant feeding practices, infant´s age, socioeconomic status and city. [file 13006_2022_455_MOESM1_ESM.docx]

**Additional file 1. Quota´s survey sample by infant feeding practices, infant´s age, socioeconomic status and city**

| **CATEGORY OF WOMEN** | **AGE OF BABY** | **SOCIO-ECONOMIC STATUS** | **MEXICO CITY** | **GUADALAJARA** |
| --- | --- | --- | --- | --- |
| Breastfeeding women | 0-12 months | Low | 25 | 25 |
|  |  | Medium | 25 | 25 |
|  |  | High | 25 | 25 |
| Women who feed their children BMS products | 0-3 months | Low | 25 | 25 |
|  |  | Medium | 25 | 25 |
|  |  | High | 25 | 25 |
|  | 4-6 months | Low | 25 | 25 |
|  |  | Medium | 25 | 25 |
|  |  | High | 25 | 25 |
|  | 7-12 months | Low | 25 | 25 |
|  |  | Medium | 25 | 25 |
|  |  | High | 25 | 25 |
|  |  | Low | 25 | 25 |
|  | 13-18 months | Medium | 25 | 25 |
|  |  | High | 25 | 25 |
