# Supplementary material for: Exposure to marketing of breastmilk substitutes in Mexican women: Sources and scope
Source: Int Breastfeed J. 2022 Mar 2;17:16. doi: 10.1186/s13006-022-00455-y (PMC8889386; doi:10.1186/s13006-022-00455-y)
Supplement: Supplementary file 2 — Additional file 2: Perceived BMS benefits categories. [file 13006_2022_455_MOESM2_ESM.docx]

**Additional file 2. -** **Perceived BMS benefits categories**

|  |  |
| --- | --- |
| **Category** | **Perceived benefit reported by the mother** |
| Convenient for mother and family | It allows me to leave the baby with someone else |
|  | It allows me to do other activities |
|  | It allows me to go to work |
|  | The baby is calmer |
|  | It´s an easy option to feed the baby |
|  | It´s easy to feed the baby out of home |
|  | It´s an option avaible facing a trouble or setback |
|  | It´s an option when I´m sick |
|  | It allows me to eat whatever I want |
|  | It easy to prepare |
|  | It´s a quick option |
|  | It helped me to set schedules |
|  | The couple is involved in feeding the baby |
|  | It helped me to establish schedules |
|  | The cost is affordable |
| Perceived benefits for the baby | The baby improves his/her health/doesn't get sick |
|  | The baby sleeps better |
|  | The baby improves its mental development |
|  | It strengthens the baby's bones |
|  | Baby makes it´s poop better/don´t constipated stomach |
|  | It´s good for the babys stomach |
|  | It´s better the baby´s development |
|  | It´s similar to breast milk |
|  | It doesn´t cause colic |
|  | The baby takes the bottle by its own |
| Perception of insufficient milk | It´s an option when you don´t have breast milk |
|  | It has vitamins |
|  | It has nutrients |
|  | It has calcium |
|  | It provides iron |
|  | It has minerals |
|  | It provides protein |
|  | It helps the baby to grow strong |
|  | The baby gains weight/weight gains The baby fills up/gets satisfied |
|  |  |
| None | None |
| Do not known | Do not known |
